# Effect and Mechanism of Yisui Fuyongtang (YSFYT) Decoction on Cognitive Function and Synaptic Plasticity in Rats with Vascular Cognitive Impairment

**DOI:** 10.1155/2022/1709360

**Published:** 2022-07-08

**Authors:** Tingliang Gong, Zhaoliang Luo, Li Huang, Caixian Xiao, Junlu Yi, Junfeng Yan, Qian Chen, Weihong Li, Wenqiang Tao

**Affiliations:** ^1^Department of Brain Disease, Chongqing Hospital of Traditional Chinese Medicine, Chongqing, China; ^2^School of Basic Medicine, Chengdu University of Traditional Chinese Medicine, Chengdu, China

## Abstract

Vascular cognitive impairment (VCI) has emerged as the second major disease responsible for dementia, and there is still a lack of effective treatment methods for this disorder to date. Clinical medications have found that Yisui Fuyongtang (YSFYT) Decoction is effective in improving neurological signs and learning-memory functions in patients who develop white matter lesions and whole brain atrophy. To clarify the effect and molecular regulation mechanism of YSFYT Decoction on model rats, this research analyzed the influence of YSFYT Decoction on the learning-memory ability and lipid metabolism of rats based on behavioral and biochemical analysis. Further pathology and protein detection methods were adopted to investigate the action of YSFYT Decoction on the neurons in the hippocampus of model rats and the regulation of the brain derived neurotrophic factor (BDNF)-tyrosine protein kinase receptor B (TrkB) signaling pathway. Compared with the VCI group, after YSFYT Decoction administration, the ratio of swimming time in the platform, number of crossing the platform, number of active avoidance, and proportion of active avoidance of the rats were markedly increased, whereas the response latency was substantially reduced (*p* < 0.05). Biochemical tests indicated that contents of lipoprotein lipase (LPL), triglyceride (TG), total cholesterol (TC), low-density lipoprotein cholesterol (LDL-C), and high-density lipoprotein cholesterol (HDL-C) of the model rats in YSFYT Decoction treatment group were greatly reduced, whereas those of total antioxidant capacity (T-AOC), glutathione peroxidase (GSH-PX), catalase (CAT), malondialdehyde (MDA), and superoxide dismutase (SOD) were elevated (*p* < 0.05). Additionally, Bcl-2 expression in YSFYT Decoction treatment group was significantly increased, but neuron apoptosis of the hippocampus tissue was reduced. Meanwhile, neuron number was apparently higher than that in VCI model group. Following Yisui Decoction treatment, expressions of growth-associated protein 43 (GAP43), synaptophysin (SYP), postsynaptic density 95 (PSD95), NMDAR subunit 2B (NR2B), BDNF, TrkB, phospho-mitogen-activated protein kinase (p-MAPK), extracellular signal-regulated kinase (ERK), phosphatidylinositol 3-kinase (PI3K), and phospho-protein kinase B (p-AKT) were markedly elevated. Taken together, YSFYT Decoction could activate the BDNF-TrkB signaling pathway, elevate Bcl-2 expression, and minimize neuronal apoptosis in hippocampus, thereby improving the behavioral characteristics and biochemical indicators of the VCI rat model.

## 1. Introduction

Vascular cognitive impairment (VCI) is various degrees of cognitive disorder and dementia that can be attributed to cerebrovascular diseases, including non-dementia VCI, severe vascular dementia, and Alzheimer's disease accompanying vascular disorder [1]. VCI is characterized by continuous declining cognitive functions. The main clinical manifestations include hypophrenia, memory deterioration, and a certain degree of speech apraxia and personality changes at varying degrees accompanying in some cases [2, 3]. The area of the brain that is mainly responsible for long-term memory of storage, conversion, orientation, and learning is called the hippocampus. The small and curved hippocampus lies between the thalamus and the inner region of the temporal lobe of the cerebrum, which belongs to partial of the cerebral limbic system. Multiple studies have confirmed that cerebral ischemia and hypoxia directly affect the pyramidal cells in the CA1 and CA3 regions of the hippocampus in rats, whereas the dentate granule cells in the hippocampus are not sensitive to such conditions [4, 5]. Additional studies have also revealed that the neurotransmitter glutamate increases markedly after cerebral ischemia and reperfusion, which can damage pyramidal cells in the CA1 region of the hippocampus of rats [6, 7]. Neuronal cell apoptosis is a primary pathological manifestation after cerebral ischemia. The gene family B-cell lymphoma-2 (Bcl-2) is mostly responsible for apoptosis regulation, and apoptosis marks an essential cause of hippocampal atrophy. The failure to induce long-term potentiation in the CA1 region of the rat hippocampus is also intimately correlated with changes in the expression levels of Bcl-2 and Bax [8, 9].

Synaptic plasticity is the activity-dependent modulation of the strength of synaptic connections, providing an essential component for learning-memory [10]. Synaptophysin (SYP) and growth-associated protein 43 (GAP43) are mainly located in the presynaptic membrane, participating in neural development and axonal regeneration. Moreover, they play important roles in the formation of synapses, the establishment, and the reconstruction of synaptic connections [11, 12]. Postsynaptic density (PSD) and microtubule-associated protein-2 (MAP-2) are found in the postsynaptic membrane and play active roles in regulating the secretion and accumulation of neurotransmitters, which are closely related to learning-memory [13]. PSD-95, an important component of PSD, regulates cellular protein expression and enzyme activity by binding to the C-terminal of N-methyl-D-aspartic acid receptor (NMDAR) and participates in the plasticity regulation of excitatory synapses [14]. NMDAR is also involved in hippocampal synaptic plasticity and learning-memory, especially its subunit NR2B, which is critical to long-term potentiation (LTP) and long-term depression (LTD) [15].Brain-derived neurotrophic factor (BDNF) plays a role in regulation in the central nervous system, and it is one of the key members of the nerve growth factor family. It is mainly distributed in the regions of hippocampus and prefrontal cortex and participates extensively in the development of neurons, synaptic plasticity, learning-memory, and emotion regulation [16, 17]. Tyrosine protein kinase receptor B (TrkB) is a neurotrophic factor high-affinity receptor encoded by tyrosine protein kinase. The BDNF-TrkB signaling pathway is regarded as a key pathway that mediates LTP to form memory. The downstream signaling pathways of BDNF consist of mitogen-activated protein kinase (MAPK) and phosphoinositide-3-kinase (PI3K) signaling pathways [18, 19]. These pathways are positively correlated with synaptic plasticity regulation, long-term potentiation, and repair and maintenance of the survival of damaged neurons.

The Yisui Fuyongtang (YSFYT) Decoction is an empirical formula of the Department of Brain Diseases of Chongqing Traditional Chinese Medicine Hospital. The formulation consists of 20 Chinese medicines including *Radix Rehmanniae Praeparata* (30 g), *Cornus officinalis* (30 g), *Radix Astragali* (30-60 g), *Codonopsis pilosula* (30 g), *Psoralea corylifolia* (10 g), *Fructus Alpiniae oxyphyllae* (30 g), *Salt Cuscutae* (15 g), *Morinda officinalis* (10 g), oyster (20 g), *Radix Salviae Miltiorrhizae* (10 g), whole scorpion (5 g), *Bombyx batryticatus* (10 g), centipede (1-2), *Schisandra chinensis* (10 g), *Polygala tenuifolia* (10 g), *Cervus nippon Temminck* (10 g), vinegar tortoise shell (30 g), and *Fructus Amomi* (10 g). A few clinical cases have indicated that YSFYT Decoction can fortify the learning-memory of patients with white matter lesions. The traditional Chinese medicines formula can exert multitargeted combination treatment through synergistic effects of drugs. In addition to the beneficial effects of antioxidation, anti-inflammation, lipid metabolism, and neuroprotection on cognitive function, the drugs *Cornus officinalis*, *Radix Astragali*, oyster, and *Schisandra chinensis* of YSFYT Decoction are also effective in minimizing cognitive impairment in improving learning-memory by enhancing neuronal synaptic plasticity, which have been confirmed by some researchers [20]. The combined use of these drugs can improve the overall therapeutic effect through the synergistic effects of drugs while treating the disease based on traditional Chinese medicine syndrome differentiation. Collectively, we speculated that YSFYT Decoction may be synergistic among the drugs and improve the treatment effectiveness, and the mechanism of action may be related to the improvement of hippocampal synaptic plasticity. The present study applied YSFYT Decoction to intervene the VCI rat model and aimed to observe and analyze the effects of YSFYT Decoction on the learning-memory ability, lipid metabolism, and oxidative stress index of the model rats using behavioral and biochemical methods. Furthermore, pathological and molecular biological detection methods were adopted to clarify relevant effects of YSFYT Decoction on synaptic plasticity in model rats. As far as we know, this study is the first to investigate the ameliorative effect of YSFYT on VCI through synaptic plasticity. We believe that exploring the potential mechanism of YSFYT decoction in improving learning-memory and synaptic plasticity disorders in model rats based on BDNF-TrkB signaling pathway can provide novel insights and research basis for solving clinical difficulties.

## 2. Materials and Methods

### 2.1. VCI Model Establishment

**Figure 1:**
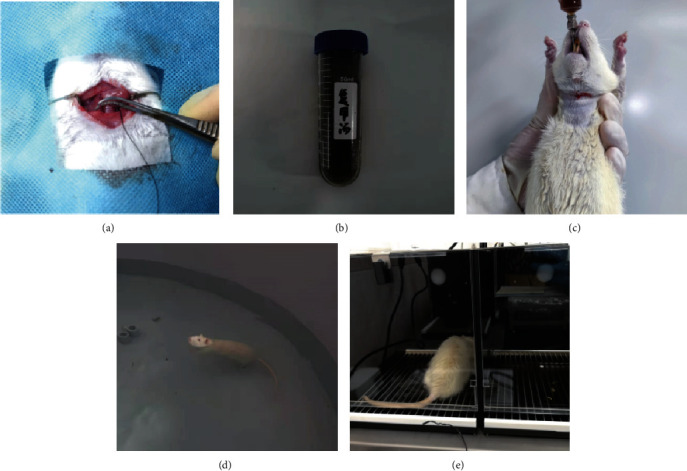
VCI rat modeling and behavioral detection. (a) VCI model preparation using the modified Pulsinelli's four-vessel occlusion method. (b)–(c) Intragastric administration. (d) Morris water maze experiment. (e) Shuttle box experiment.

A total of 63 five-week-old male SD rats (13 standby) weighing 180-200 g were provided by Chongqing Ensiweier Biotechnology Co., Ltd. After seven-day adaptive feeding, all rats were classified into 5 groups randomly: sham operation group; VCI group, high-dose YSFYT Decoction group, low-dose YSFYT Decoction group, and aniracetam group (positive control). Aniracetam, an analog of piracetam, is used for enhancing cognition and improving memory under conditions when the brain has been damaged. Each group had ten rats, and they were kept under standard conditions of light (12/12 h light/dark cycle), temperature (23-25 °C), and humidity (40-60%) with food and water ad libitum. All the rats were anesthetized with 2% sodium pentobarbital, 0.3 mL/100 g. Except for the sham group, the rest repeated the VCI model using the modified Pulsinelli four-vessel occlusion method. The procedure course of the sham operation group was the same as that of the VCI group, but the vertebral artery was not thermocoagulated and failure in clamping the common carotid artery (Figure 1(a)). After the model was successfully established, intragastric administration was started, and the dosage administered was based on a converted dose of a 200 g rat equivalent to the mass of a 70 kg adult (Figures 1(b)–1(c)) [21, 22].

In the high-dose YSFYT Decoction group, as per conversion coefficient 6.3 of the dose between rats and humans, the clinical dose of intragastric administration was determined twice that of a human equivalent dose, containing 54 g/kg of crude drug, for 1 week.

In the low-dose YSFYT Decoction group, as per conversion coefficient 6.3 of the dose between rats and humans, the clinical dose of intragastric administration was determined the same to a human equivalent dose, containing 27 g/kg of crude drug, for 1 week.

In the aniracetam group, as per conversion coefficient 6.3 of the dose between rats and humans, the clinical dose of intragastric administration was 0.0625 g/kg, for 1 week.

In the sham operation group and model control group, An equal amount of normal saline was intragastrically administered for 1 week.

Following continuous administration for 1 week (normal saline was given in both sham and VCI groups), Morris water maze experiment and shuttle box experiment were performed, respectively. All rats were then sacrificed, and the peripheral blood sample, temporal lobe, and hippocampus tissues of the rats were collected for detection of related indicators.

### 2.2. Morris Water Maze Experiment [23]

The experiment was carried out in two parts; the first part was directional navigation training. The rats were placed in the water maze 2 h before training to get familiar with the environment. Upon training, each rat of the experimental groups was placed at the same site in the water facing the pool wall every day, and the time that the rats required from entry into the water to climbing on the underwater platform within 120 s (escape latency) was documented. The rats were guided to the platform if they failed to locate within 120 s training and kept there for 30 s. And latency was considered to be 120 s. The rats stayed on the platform for 10 s if they found it during the training to fortify their memory. Directional navigation training was conducted twice a day for 5 consecutive days. On day 6, e.g. 24 h after the completion of the training, the platform was removed, and the spatial learning was started. The first time period of rats crossing the original platform quadrant (target quadrant) after entering water, the number of times crossing the original platform quadrant within 2 min, and the time spent on the original platform quadrant were documented. According to the obtained data, the spatial learning ability and memory of rats in each group were assessed. Shorter latency in positioning the platform, shorter first time to cross the platform, larger number of times to cross the platform, and longer time to stay in the target quadrant indicated that the tested rats had better spatial learning and memory capabilities, and vice versa (Figure 1(d)).

### 2.3. Shuttle Box Test [24]

The test box (50 cm ×16 cm ×10 cm) was divided into two parts: a light compartment and a dark compartment, with holes between the two. There was a copper grid available for electricity at the bottom of the dark compartment with a current of 0.5 mA. The experiment was completed in 3 days. On the first day, the rats were put into the light compartment with their heads towards outside allowing to move freely for 4 min. On day 2, the animals were placed in the light chamber again, with an open gate between both compartments after 60 s free movement in the light compartment. Once the animals were in the dark chamber, the gate between the light and dark chambers was closed, and an electric shock of 0.5 mA was given for 3 s to stimulate the memory. Following 24 h after the electric shock, the following data were recorded: The number of electric shocks (referred to the number of passive avoidance; the difference between this number and the set number of cycles was the number of active avoidance); stimulating time (referred to passive avoidance latency); and active avoidance response time (referred to active avoidance latency) (Figure 1(e)).

### 2.4. Detection of Lipid Metabolism and Oxidative Stress Indicators

The animals were fasted for 12 h, and water drinking was forbidden for 2 h before blood collection. Rats were anesthetized with sodium pentobarbital and fixed in supine position, and the abdominal cavity was cut through the skin of the ventral median line to expose the abdominal aorta; then, blood was extracted with a syringe. The blood biochemical analyzer was employed to detect the lipoprotein lipase (LPL), total cholesterol (TC), triglyceride (TG), low-density lipoprotein cholesterol (LDL-C), and high-density lipoprotein cholesterol (HDL-C) in each group of rats.

ELISA was performed to determine the contents of catalase (CAT), malondialdehyde (MDA), glutathione peroxidase (GSH-PX), total antioxidant capacity (T-AOC), and superoxide dismutase (SOD) in hippocampus. ELISA kit was purchased from Quanzhou Ruixin Biotechnology Co., Ltd. The plates required were firstly balanced at room temperature for 20 min and taken from an aluminum foil bag. Additional plates placed back after being sealed in a self-sealing bag and stored at 4 °C. Standard and sample wells were prepared firstly. To each standard well, 50 *μ*L standard at several concentrations was supplemented. To each sample well, 50 *μ*L sample to be tested was supplemented, and the blank wells were left untreated. Except the blank wells, 100 *μ*L detection antibody labeled by horseradish peroxidase (HRP) was supplemented to both standard and sample wells, respectively. Following blocked with a sealing membrane, the samples were incubated at a 37 °C water bath or a constant thermostat for 60 min. Following removal of liquid, the samples were dried on a piece of absorbent paper, and the wells were supplemented and full of washing solution (350 *μ*L), left undisturbed for 1 min, discarded the medium, dried again on a pieced of absorbent paper, and repeated 5 times. Following addition of substrates A and B 50 *μ*L, respectively, to each well, incubation was performed at 37 °C for 15 min in dark. Termination solution at a quantity of 50 *μ*L was supplied to the wells and the OD values of which were measured within 15 min at 450 nm wavelength.

### 2.5. HE Staining

The specimens were collected and then washed using PBS, fixed using 4% paraformaldehyde, and followed by being soaked in 70%, 80%, 90%, 95%, and 100% ethanol for 1.5 h, respectively. After dehydration, the tissues were immersed and kept in xylene for 20 min. The tissues were under transparency treatment, paraffin embedded for 3 h, prepared into blocks, and sectioned at 2.5 *μ*m thickness. The sections were placed on adherent slides and incubated in an oven at 55 °C, ensuring tight adherence to the slides. The embedded tissues were then immersed in xylene I and II for dewaxing 10 min, respectively, and then soaked in 100% ethanol I, 100% ethanol II, 95% ethanol I, 95% ethanol II, 85% ethanol, and 75% ethanol for 3 min, respectively. Following three cycles of washing using double distilled water, 2 min each time, the sections were dyed using hematoxylin for 3 min, rinsed the excess staining solution with tap water for about 3 min, and followed by a cycle of washing using distilled water. Section destaining was performed with 1% hydrochloric acid alcohol to remove excess hematoxylin staining solution in the cytoplasm and followed by eosin staining for 1 min.

The excess dye solution was rinsed using tap water for about 3 min. Dehydration was performed using ethanol at 75% and 95%, 10 s each, and finally anhydrous ethanol for 1 min. Xylene was employed for transparency for 5 min, and the slides were fixed with neutral resin. Photography of the sections employed an Mshot MF53 microscope produced by Guangzhou Mingmei Optoelectronic Technology Co., Ltd.

### 2.6. Nissl Staining

The paraffin sections were dewaxed firstly using xylene I and II, processed using 100% ethanol I and II, 95% ethanol I and II, and 85% and 75% ethanol, respectively, 3 min each time, followed by three times of rinsing using double distilled water, 2 min each time. The sections were put into Cresyl Violet Stain, heated until steaming with fire, and cleaned using distilled water for 2 s. Nissl differentiation was performed immediately for observation under a microscope until the background faded away. Anhydrous ethanol was used for quick dehydration. The slides were transparent using xylene and sealed with neutral resin. Microscopic examination revealed purple Nissl substances with a background close to colorless or light blue. The sections were photographed the same as previously described.

### 2.7. TUNEL Staining

The samples were dewaxed twice using xylene, 15 min each time. Anhydrous ethanol was employed then and for 5 min, followed by 90% ethanol for 2 min, 70% 2 min, and distilled water 2 min. The samples were subsequently supplemented with 20 *μ*g/mL DNasc-free protease K, treated at 37 °C for 30 min, and washed 3 cycles using PBS or PBSS. During the preparation of the TUNEL detection solution: TdT enzyme:fluorescent-tagged solution was 1 : 9. The detection solution was dropped and incubated at 37 °C away from light. Following three cycles of washing using PBS, DAPI nucleus staining was carried out and lasted for 5 min. The slides were washed three cycles using PBS or PBSS, sealed using anti-fluorescence quenching solution, and ultimately visualized under a fluorescence microscope.

### 2.8. Immunohistochemistry

The EDTA-sodium citrate antigen repair solution was boiled for 30 min, naturally cooled to room temperature, and followed by PBS washing 3 times, 5 min each time. Subsequently, 3% hydrogen peroxide was employed for treatment at room temperature for 15 min and cleaned 3 times, 5 min each. The sections were incubated at room temperature blocked by goat serum 30 min, removed the blocking solution (no bleaching), supplied with primary antibodies for incubation at 4 °C overnight, and rinsed 3 times using PBS, 5 min each time. Secondary antibody working solution (HRP labeled) was supplied for incubation at room temperature for 1.5 h. The tissues were cleaned 3 times using PBS, 5 min each time. DAB development was performed avoiding light for 5 min and then rinsed twice using distilled water for 5 min each, hematoxylin restaining for 2 min, tap water washing for blue returning, 1% hydrochloric acid ethanol differentiation for 5 s, and 95% ethanol dehydration for 2 min. Fresh 95% ethanol was employed for dehydration another 2 min, and xylene was employed for transparency for 5 min. Fresh xylene was used for transparency for additional 5 min, and the slides were fixed with neutral resin. Photography of the sections were conducted via an inverted Mshot MF53 microscope (Guangzhou Mingmei, China).

### 2.9. qPCR Detection

Trizol method was used to extract total RNA, and T5 Fast qPCR kit was used for qPCR test. The reaction system included 2 × T5 Fast qPCR Mix, 10.0 *μ*L; 10 *μ*M Primer F, 0.8 *μ*L; 10 *μ*M Primer R, 0.8 *μ*L; 50 × ROX Reference Dye II, 0.4 *μ*L; Template DNA, 0.5 *μ*L; and 7.5 *μ*L ddH2O. Reaction conditions included 95 °C 30 s, 95 °C 5 s, 55 °C 30 s, 72 °C 30 s, and 40 cycles in total. Primer sequences used are presented in Table 1: GAPDH, AGGTCGGTGTGAACGGATTTG/TGTAGACCATGTAGTTGAGGTCA.

### 2.10. Western Blot Detection

Precooled RIPA lysate was added for lysis on ice for 15 min and vortex shaking for 30 s and followed by ice lysis for another 15 min. The tissues were centrifuged at 12 000 r/min 4 °C for 10 min for supernatant collection. After quantification using bicinchoninic acid (BCA) kit, PBS and sample loading buffer were supplemented to set the final protein concentration at 2.5 *μ*g/*μ*L. The protein samples were firstly incubated at 100 °C, denatured 5 min, and stored at −80 °C. Electrophoresis was subsequently performed using 10% SDS-PAGE gel. Concentrated gel was under 80 V constant voltage for 30 min, and separate gel was at 120 V constant voltage for 90 min. The protein was transferred to a PVDF membrane under the condition of 70 V constant voltage for wet transfer 90 min. After membrane transfer, TBST solution containing 5% skim milk powder was employed for blocking 1 h at room temperature. The membrane was provided with primary antibody and incubated with mild vibration overnight at 4 °C. Primary antibodies of GAP43 (A19055, 1 : 1000), MAP-2 (A17409, 1 : 1000), SYP (A19122, 1 : 1,000), PSD95 (A0131, 1 : 1000), NR2B (A3056, 1 : 5000), BDNF (A4873, 1 : 1000), TrkB (A19832, 1 : 1000), MAPK (A5049, 1 : 1000), p-MAPK (AP0526, 1 : 1000), ERK (A19630, 1 : 1000), PI3K (A19742. 1 : 1000), AKT (A17909, 1 : 1000), p-AKT (AP1259, 1 : 1000), and GAPDH (A19056, 1 : 10000) were purchased from ABclonal Technology, Wuhan, China. The membrane was washed 3 times with TBST, 10 min each time, the following day. After secondary antibody was added for incubation for 2 h, an ultrasensitive luminescence color developing solution was added for incubation for 1 min and then developed in a gel imaging system. ImageJ software was employed for gray value analysis of images.

### 2.11. Statistical Analysis

Statistical software Graphpad Prism 8.0 (Graphpad, USA) was utilized for data analysis. Measurement data were expressed by mean ± SEM. *p* < 0.05 was considered statistically significant.

## 3. Results

### 3.1. Yisui Fuyongtang Decoction Can Effectively Improve the Behavioral Characteristics of VCI Model Rats

**Figure 2:**
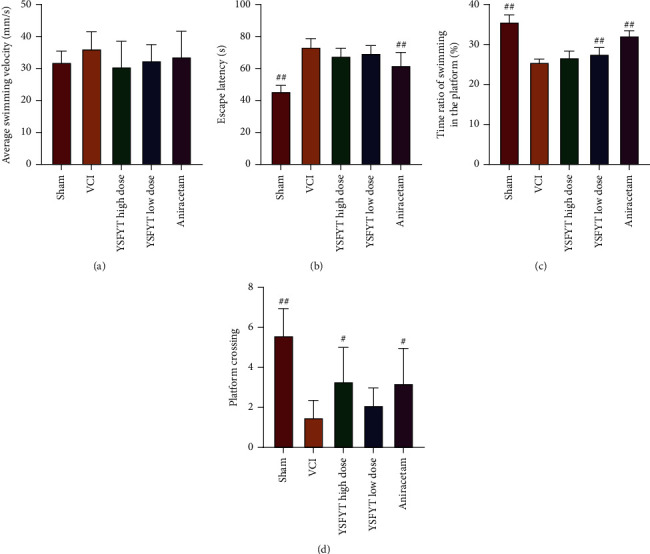
Morris water maze results. (a) Average swimming velocity. (b) Escape latency. (c) Ratio of swimming time in plateau period. (d) Four indicators of the number of crossing platforms. ^#^*p* < 0.05; ^##^*p* < 0.01.

We subsequently performed Morris water maze tests and shuttle box tests; the behavioral changes of rats after administration were detected to determine the therapeutic and protective effects of YSFYT Decoction on VCI rats. Morris water maze test detected average swimming velocity, escape latency, ratio of platform swimming time, and number of crossing platform. As shown in Figure 2, escape latency time of the VCI model group was markedly increased, and the time ratio of platform swimming and number of crossing platform were substantially decreased (*p* < 0.01) compared with the sham operation group, indicating that VCI model rats had developed cognitive dysfunction. Compared with the VCI group after low-dose YSFYT Decoction administration, the time ratio of platform swimming was markedly elevated (*p* < 0.01) (Figure 2(c)). Compared with the VCI group, the number of platform crossing was significantly increased after high-dose YSFYT Decoction administration (*p* < 0.05) (Figure 2(d)).

**Figure 3:**
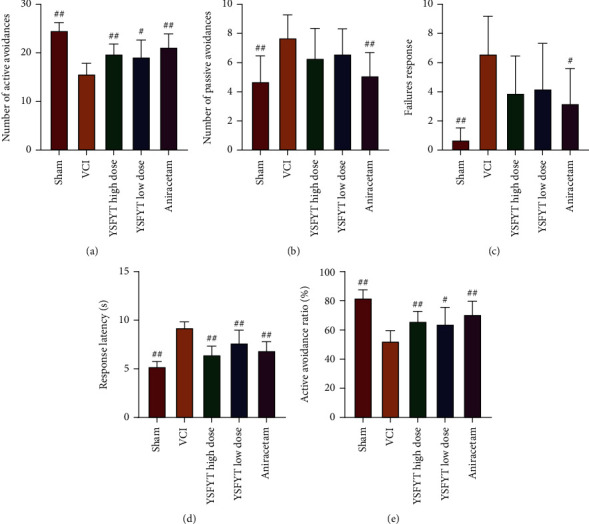
Shuttle box experiment results. (a) Number of active avoidance. (b) Number of passive avoidance. (c) Escape response failures. (d) Response latency. (e) Proportion of active avoidance. ^#^*p* < 0.05; ^##^*p* < 0.01.

The shuttle box detected numbers of active and passive avoidance, number of avoidance failures, stimulus latency, and ratio of active avoidance. The number of passive avoidance, the number of avoidance failures, and the response latency of rats in the VCI group were markedly increased (*p* < 0.01) versus sham operation group, whereas the number and ratio of active avoidance were greatly decreased (*p* < 0.01), indicating that rats of VCI group had apparent cognitive dysfunction (Figure 3). After YSFYT Decoction administration, the number of active avoidance and the ratio of active avoidance of the rats in both high and low doses of YSFYT Decoction groups were markedly increased (*p* < 0.05) (Figures 3(a) and 3(e)), whereas the response latency was substantially reduced (*p* < 0.01) versus VCI group (Figure 3(d)). Generally, these previously described findings indicated that YSFYT Decoction could effectively improve the behavioral characteristics of VCI model rats.

### 3.2. YSFYT Decoction Can Effectively Improve the Biochemical Indexes of VCI Model Rats

**Figure 4:**
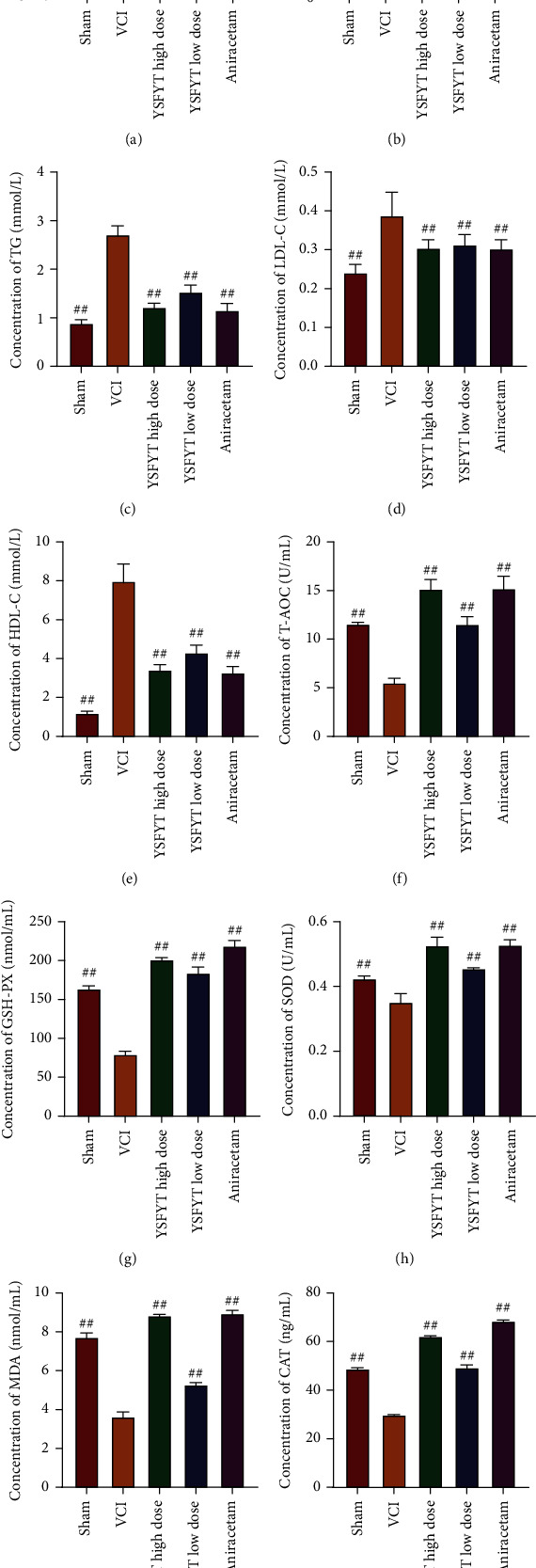
Biochemical index detection of rats. (a–e) LPL, TC, TG, LDL-C, and HDL-C detection of serum by blood biochemical analyzer. (f–j) T-AOC, GSH-PX, CAT, MDA, and SOD detection in hippocampus by ELISA. ^##^*p* < 0.01.

After the behavioral study, peripheral blood of rats was collected, and the concentrations of LPL, TC, TG, LDL-C, and HDL-C of rats in each group were identified using an automatic biochemical analysis system. The results revealed that LPL of sham operation group, VCI group, high-dose group, low-dose group, and positive control group were 1.51 ± 0.02, 3.11 ± 0.05, 1.76 ± 0.04, 2.75 ± 0.02, and 1.78 ± 0.03 mmol/L, respectively. The TC concentrations in the mentioned groups were 1.35 ± 0.05, 5.90 ± 0.18, 2.50 ± 0.09, 3.12 ± 0.20, and 2.80 ± 0.10 mmol/L, respectively. The TG concentrations were 0.89 ± 0.02, 2.71 ± 0.06, 1.22 ± 0.03, 1.53 ± 0.04, and 1.16 ± 0.04 mmol/L, respectively. The LDL-C were 0.24 ± 0.01, 0.39 ± 0.02, 0.31 ± 0.01, 0.31 ± 0.01, and 0.30 ± 0.10 mmol/L, respectively. The HDL-C were 1.21 ± 0.03, 7.98 ± 0.28, 3.43 ± 0.08, 4.31 ± 0.12, and 3.28 ± 0.10 mmol/L, respectively (Figures 4(a)–4(e)). Generally, in contrast to sham operation, contents of LPL, TC, TG, LDL-C, and HDL-C in the VCI group were greatly elevated. Conversely contents of the described indexes were markedly reduced in the treatment groups administered with YSFYT Decoction versus VCI group.

ELISA was performed to determine the contents of CAT, MDA, T-AOC, GSH-PX, and SOD in hippocampus. The results showed that T-AOC contents of the sham operation group, VCI group, high-dose group, low-dose group, and positive control group were 11.57 ± 0.09, 5.51 ± 0.23, 15.17 ± 0.48, 11.55 ± 0.38, and 315.22 ± 0.62, respectively. The GSH-PX contents of each group were 163.9 ± 1.89, 79.46 ± 2.00, 201.3 ± 1.15, 184.3 ± 3.67, and 219 ± 3.30 nmol/mL, respectively. The CAT contents of each group were 48.87 ± 0.17, 29.87 ± 0.06, 62.21 ± 0.12, 49.34 ± 0.51, and 68.5 ± 0.21 ng/mL, respectively. The MDA contents of each group were 7.73 ± 0.11, 3.64 ± 0.12, 8.85 ± 0.03, 5.29 ± 0.05, and 68.95 ± 0.08 nmol/mL, respectively. The SOD contents of each group were 0.43 ± 0.00, 0.35 ± 0.01, 0.53 ± 0.01, 0.46 ± 0.00, and 0.53 ± 0.01 U/mL, respectively (Figures 4(f)–4(j)). Generally, the contents of LPL, TC, TG, LDL-C, and HDL-C in the VCI group were greatly reduced versus the sham operation group. Compared with the VCI group, the contents of the described indexes were markedly elevated in the treatment groups administered with YSFYT Decoction.

### 3.3. YSFYT Decoction Effectively Increases the Number of Hippocampal Neurons

**Figure 5:**
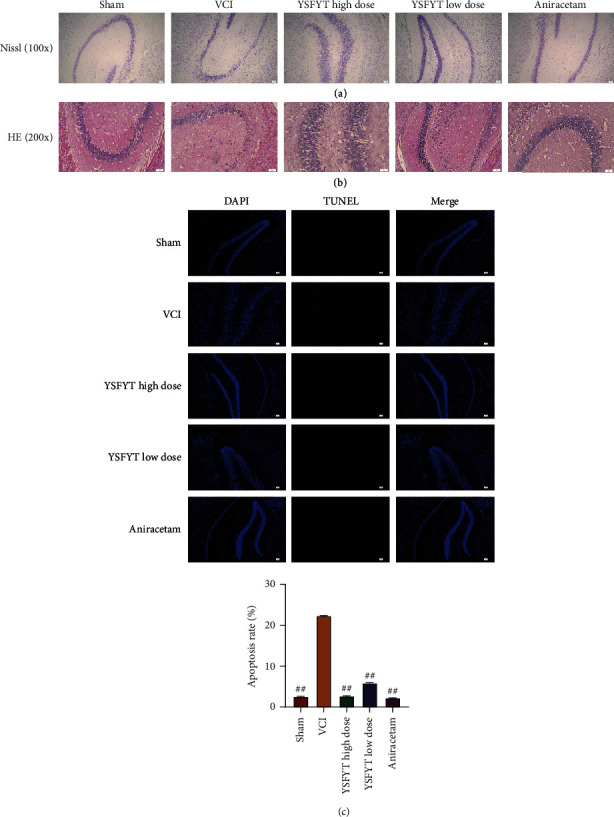
Histological examination of hippocampal neurons. (a) Nissl staining. (b) HE staining. (c) Fluorescence TUNEL assay. The scale bar is 50 *μ*m.

Next, we separated and sliced the hippocampal tissues of rats in all groups and visualized changes in the number of Nissl bodies in the hippocampus using Nissl and HE staining (Figures 5(a)–5(b)).  Figure 5 presented that the number of Nissl bodies in the VCI group was less than that in the sham operation group under the same view field. Compared with VCI group, the number of Nissl body was significantly increased in the hippocampus of both YSFYT and aniracetam positive drug groups. Moreover, fluorescent TUNEL assay was conducted to determine cell apoptosis of hippocampal tissues. The findings revealed that the percentage of apoptotic cells in sham operation, VCI, YSFYT Decoction high-dose, YSFYT Decoction low-dose, and aniracetam groups were 2.56%, 22.34%, 2.69%, 5.88%, and 2.23%, respectively (Figure 5(c)).

The above described results indicated that apoptosis of hippocampal neurons was elevated in VCI model rats, whereas the number of neuronal cells decreased. YSFYT Decoction could effectively reduce apoptosis of hippocampal neurons while increase the number of neurons in model rats.

**Figure 6:**
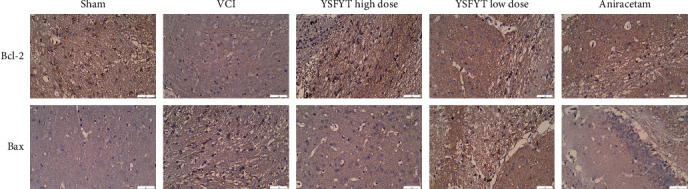
Immunohistochemistry was used to detect the expressions of antiapoptotic protein Bcl-2 and proapoptotic protein Bax in hippocampus of each group. Brown indicates the positive part. The scale bar is 50 *μ*m.

Furthermore, we used immunohistochemistry to detect the expressions of antiapoptotic protein Bcl-2 and proapoptotic protein Bax in the hippocampus of each group, indicating that the expression of Bcl-2 in the VCI group (brown indicated positive parts) decreased apparently, and the expression of Bax was markedly increased compared with sham operation group. The positive rates of Bcl-2 in the high-dose and low-dose YSFYT Decoction groups were substantially higher than that of the VCI group, indicating that YSFYT Decoction might regulate neuronal cell apoptosis by affecting apoptosis-related proteins (Figure 6).

### 3.4. Molecular Regulatory Mechanism of YSFYT Decoction

**Figure 7:**
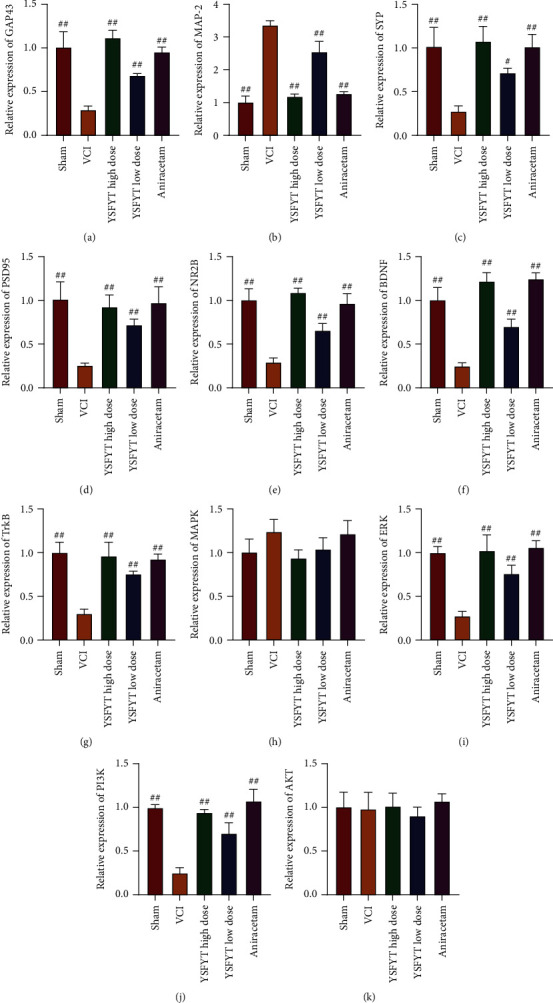
Detection of gene expressions by qPCR. (a–k) The mRNA expression levels of GAP43, MAP-2, SYP, PSD95, NR2B, BDNF, TrkB, MAPK, ERK, PI3K, and AKT. ^#^*p* < 0.05; ^##^*p* < 0.01.

**Figure 8:**
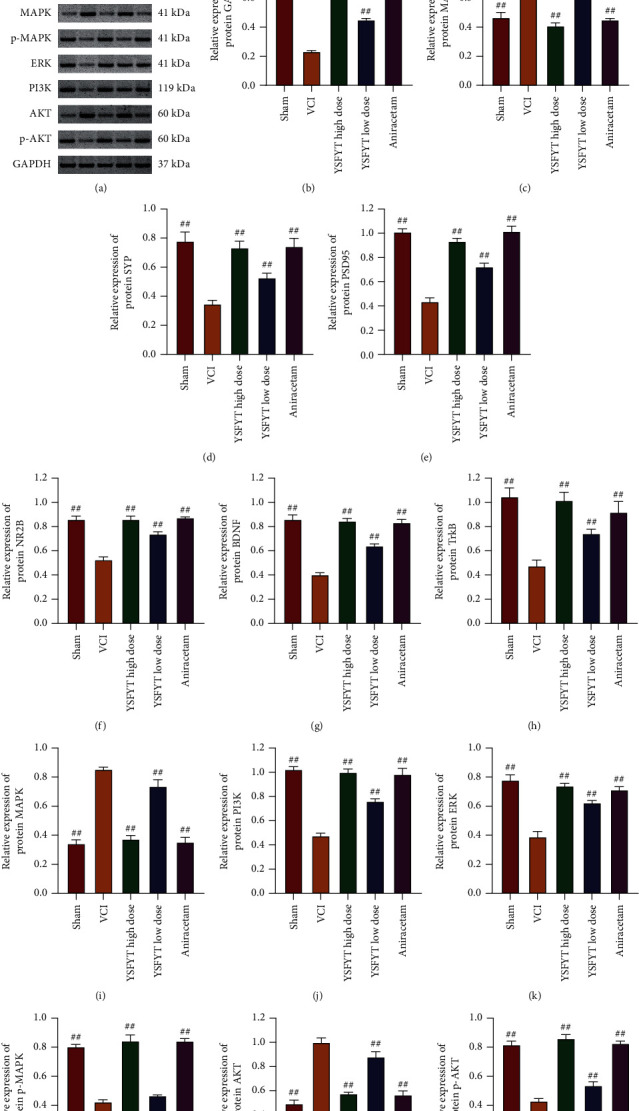
Western blot detection of protein expressions. (a) Results of protein bands. (b–n) Relative protein expressions of GAP43, MAP-2, SYP, PSD95, NR2B, BDNF, TrkB, MAPK, p-MAPK, ERK, PI3K, AKT, and p-AKT. ^#^*p* < 0.05; ^##^*p* < 0.01.

We finally detected the expression of VCI-related signaling pathway molecules. mRNA expression levels of GAP43, MAP-2, SYP, PSD95, NR2B, BDNF, TrkB, MAPK, ERK, PI3K, and AKT were determined using qPCR, and their protein levels were identified using Western blot as well. The qPCR assays revealed that expressions of GAP43, SYP, PSD95, NR2B, BDNF, TrkB, ERK, and PI3K in the VCI model group were greatly reduced, whereas MAP-2 expression was apparently elevated versus sham operation group. YSFYT Decoction treatment could reverse the expression changes of described genes in the VCI model group (Figure 7). The mRNA expression levels of MAPK and AKT did not change significantly in each group. Western blot findings indicated that the expression levels of GAP43, SYP, PSD95, NR2B, BDNF, TrkB, p-MAPK, ERK, PI3K, and p-AKT in the VCI model group were significantly reduced, whereas the expressions of MAP-2, MAPK, and AKT were greatly increased. Similarly, in the YSFYT Decoction treatment group, the changes in protein expression in the VCI group were restored to a certain extent (Figure 8). The previously obtained results indicated that YSFYT Decoction could restore the differential expression of functional gene expression caused by VCI.

## 4. Discussion

As VCI has a short life expectancy, it impairs the social function and even motor function of the patient after onset, resulting in an apparent decline in quality of life for patients. It is therefore that this disease has gained increasing attention in recent years [25]. For VCI research, one prerequisite is to construct an animal model that is consistent with the disease, through which the pathogenesis, pathological changes, and how to effectively intervene early VCI can be investigated. In VCI model preparation, rodents are the most widely applied. The preparation methods of VCI model include chronic whole cerebral hypoperfusion, focal hypoperfusion, unilateral middle cerebral artery occlusion and reperfusion, vascular embolization appeasement, and whole cerebral ischemia (i.e. modified Pulsinelli four-vessel occlusion method). As well as some comorbidities based on cerebrovascular diseases, such as hyperlipidemia, spontaneously hepertensive rats, homocysteineblood syndrome, Notch3 mutation, optically induction, and intracerebral amyloidosis,can be used to prepare VCI model. Pulsinelli four-vessel occlusion is the most reliable animal model of VCI caused by chronic cerebral ischemia in recent years [26, 27]. This method can better restore the VCI pathogenesis with a tiny wound. Its advantages also include fast postoperative recovery of rats and relatively low mortality, so it is widely used in the VCI research. In this experimental study, the modified Pulsinelli four-vessel occlusion method was used. The clinical characteristic of VCI is cognitive dysfunction, so whether the VCI rat modeling is successful or whether the cognitive function of rats is damaged and whether the treatment method is effective or whether the cognitive function of the rats is improved were mainly verified using behavioral tests. At present, the common evaluation of the cognitive function of experimental animals mainly focuses on the reference memory and working memory of rats. Commonly used behavioral experiments include Morris water maze, Barnes maze, radial arm maze test, and shuttle box test. Among them, the Morris water maze is the most widely introduced one and is currently recognized as a relatively objective evaluation method for learning-memory function globally. Morris water maze behavior experiment is divided into positioning navigation experiments on the first 4 days and spatial exploration experiments on the fifth day, allowing to clarify the spatial learning-memory ability of rats. Its working principle is to force the rat to swim and locate a hidden escape platform with the survival instinct. This process involves complex memory capabilities, including collecting visual information and spatial positioning, and then reorganizing, consolidating, and re-extracting the information. The purpose is to find a hidden escape platform in the shortest distance as soon as possible. The water maze experiment is the most commonly used in behavioral neuroscience research. This experiment is considered to be related to hippocampal long-term potentiation (LTP), N-methyl-D-aspartate (NMDA), glutamate dehydrogenase, and related gene ryanodine receptor type2 (RyR2). This experiment is often used to establish rodent neurocognitive disease models and to evaluate the feasibility of neurocognitive therapy [28, 29]. In this study, we used the water maze and shuttle box tests to detect the behavioral characteristics of rats and found that the escape latency of the VCI model group was greatly increased, and the time ratio of platform swimming and the number of crossing the platform were apparently reduced compared with sham operation group. Numbers of passive avoidance, escape failure, and response latency elevated substantially (*p* < 0.01), whereas that of active avoidance and the proportion of active avoidance decreased significantly, indicating that the VCI model rats had already developed cognitive dysfunction. After YSFYT Decoction administration, the time ratio of platform swimming, and the number of crossing the platform of rats increased significantly compared with the VCI group, the number of active avoidance and the proportion of active avoidance of rats increased significantly, indicating that YSFYT Decoction effectively improved the behavioral characteristics of VCI model rats.

VCI occurs due to a lack of blood supply to the brain for various reasons and consequently leads to cerebral tissue ischemia and hypoxia. It is manifested as a chronic progressive disease of memory loss, intellectual impairment, and cognitive impairment, which can further evolve into vascular dementia (VaD) [30, 31]. Some latest research have found that the incidence of VCI is increasing but the average age of victims is decreasing. At present, discussions have been focused on pathogenesis mainly related to the excessive release of excitatory amino acids, which causes vasoconstriction and damages the blood-brain barrier, inflammation after ischemia and hypoxia, upregulation, and accumulation of inflammatory factors secondary to neuronal injury; the disorder of brain free radical balance; aggravated cerebral thrombosis caused by changes in hemodynamics; and ischemia-induced apoptosis related factor production, which can trigger neuronal necrosis and accelerate cell apoptosis and exerts an essential role in exploring the physiological and pathological mechanisms of VCI [32, 33]. It is known that when VCI develops, the antioxidant capacity of cells in the brain tissue declines substantially. Various cellular components react with free radicals in the body as a result of structural damage to vascular endothelial and smooth muscle cells. Dysfunction of cell metabolism simultaneously damages neurons, activates apoptosis-related proteins, and induces neuronal cell apoptosis. The hippocampus lies between the cerebral thalamus and the medial temporal lobe. As a part of the limbic system, it is the most sensitive cerebral region of rats to ischemia and hypoxia [34, 35]. Furthermore, this organizational structure is intimately correlated to the storage of memory information and learning functions. From the physiological anatomy level, the hippocampus area in the rat brain can be divided into two parts, the hippocampal gyrus and the dentate gyrus. The hippocampal gyrus includes four parts CA1, CA2, CA3, and CA4. It is mainly composed of pyramidal neurons. The dentate gyrus is basically composed of granular cells. Among them, the regions of CA1 and CA3 are mainly responsible for spatial discrimination and learning-memory functions. The vertebral cells in these two regions are more obviously affected by ischemia and hypoxia. Secondary cell apoptosis is the bedding for the delayed neuronal death [36, 37]. Therefore, inhibiting apoptosis of the hippocampus is an important treatment for VCI. Neuron nuclear antigen is a soluble nuclear protein, as a marker of nerve cell specific antigen. Its immunoreactivity begins to appear after the differentiation and maturity of neurons and directly participates in the development, differentiation, and functions of the nervous system. It has been widely used in the development of the nervous system, pathological diagnosis, neuronal degeneration, and necrosis. Differing from cell necrosis, apoptosis refers to the programmed cell death regulated by genes. Multiple research have confirmed that cerebral ischemia and VCI hypoxia link closely to cell apoptosis. The family of Bcl-2 gene consists of a series of proto-oncogene proteins, which serves as principal target molecules in apoptosis mechanism exploration. This family includes antiapoptotic subfamilies Bcl-XL, BclW, and Bcl-2, and proapoptotic subfamilies Bak and Bax. The Bcl-2 protein of the antiapoptotic subfamily is mainly located in the outer membrane, endoplasmic reticulum, and nuclear membrane cytoplasm of mitochondria. Its mechanism of action is to inhibit cell apoptosis by preventing Cyt-C release and blocking Caspase-3 activation. On the other hand, the Bax protein in the proapoptotic subfamily transfers to the mitochondrial membrane by itself, which increases the release of Caspase-9 and ultimately promotes cell death. Recent studies have shown that cell apoptosis mainly depends on the regulation of Bcl-2 and Bax and research on the ratios of both are more valuable than the two itself [26, 38]. A decrease in the ratio proves to promote apoptosis. On the contrary, an increase in the ratio is to inhibit apoptosis. As the most important antiapoptotic gene, Bcl-2 is also a protective substance for endogenous nerves. Its high expression can inhibit neuronal apoptosis caused by ischemia. Therefore, it is of vital importance in VCI research. The study carried out Nissl staining and HE staining to visualize changes in the number of Nissl bodies in the hippocampus. The findings indicated that the number of Nissl bodies in the VCI group was less than the sham operation group. Conversely, those in YSFYT Decoction and aniracetam positive drug groups were markedly increased compared with VCI group. Moreover, TUNEL fluorescent assays were conducted to determine cell apoptosis of hippocampal tissues and the findings revealed that percentages of apoptotic cells in sham operation, VCI, YSFYT Decoction high-dose, YSFYT Decoction low-dose, and aniracetam groups were 2.56%, 22.34%, 2.69%, 5.88%, and 2.23%, respectively. Immunohistochemistry was performed to detect the expressions of proapoptotic protein Bax and antiapoptotic protein Bcl-2 in the hippocampus of each group, indicating that Bcl-2 expression in VCI group decreased apparently, but Bax expression elevated compared with sham operation. The positive rate of Bcl-2 in the high-dose and low-dose YSFYT Decoction groups was substantially higher than VCI group, indicating that YSFYT Decoction might regulate neuronal cell apoptosis by affecting apoptosis-related proteins.

The treatment of VCI with traditional Chinese medicine is highly valued because of its minor side effects and remarkable curative effect. Recently, scholars have found that invigorating the kidney and promoting blood circulation can elevate BDNF level and receptor TrkB expression in hippocampal neurons of mice, thereby improving cognitive functions of VD mice and promoting the recovery of nerve function [39]. Wuqin Jiannao Formulation can significantly inhibit the abnormally high expression of CDK5 in the hippocampus and enhance learning-memory of dementia rats [40, 41]. Berberine hydrochloride has been reported to improve cognitive functions of rats with chronic cerebral ischemia by activating AMPK and inhibiting the expression of mTOR [42]. The present study revealed that YSFYT Decoction could improve the protein expression levels of GAP43, SYP, PSD95, NR2B, BDNF, TrkB, p-MAPK, ERK, PI3K, and p-Akt and reduce neuronal apoptosis in hippocampal tissues, thereby improving the behavioral characteristics and biochemical indicators of VCI rat model. This study provided a new research reference for the therapeutic effect and molecular mechanism of traditional Chinese medicine and offered a novel proof for the clinical treatment of VCI applying YSFYT Decoction.

## 5. Conclusions

YSFYT Decoction could activate the BDNF-TrkB signaling pathway, elevate Bcl-2 expression, and minimize neuronal apoptosis in hippocampus, thereby improving the behavioral characteristics and biochemical indicators of the VCI rat model.

## Figures and Tables

**Table 1:** Primer sequences of qPCR.

Gene name	Primes sequence (5′-3′)	
	Forward	Reverse
GAP43	CTGAGGAGGAGAAAGACGCTGTA	TGGGAGTCGCAGGAGATTTTG
MAP-2	CCATCTTGGTGCCGAGTGAG	TGGGAGTCGCAGGAGATTTTG
SYP	CTCCACTCCCCTCATCCACTA	TTCCCTCCCTGTCCTCCTTTT
PSD95	CATTGCCCTGAAGAACGC	ATGGATCTTGGCCTGAA
NR2B	CACGGTGCCTTCAGAGTT	CCTCCTCCAAGGTGACAA
BDNF	AGAGCAGCTGCCTTGATGTT	TCGTCAGACCTCTCGAACCT
Tr*κ*B	CCTCCACGGATGTTGCTGA	GGCTGTTGGTGATACCGAAGTA
MAPK	GAGAGGCCCACGTTCTACC	CGTAACCCCGTTTTTGTGTCA
ERK	TCAACCCAAACAAGCGCATCACAG	TCCAGCTCCATGTCGAAGGTGAAT
PI3K	AGCACCGACTTCAAGACTACG	GGATGCCAATGAGATTGTCC
AKT	AACGGACTTCGGGCTGTG	TTGTCCTCCAGCACCTCAGG
GAPDH	AGGTCGGTGTGAACGGATTTG	TGTAGACCATGTAGTTGAGGTCA

## Data Availability

The data used to support the findings of this study are included within the article.
